# HCMV as an Oncomodulatory Virus in Ovarian Cancer: Implications of Viral Strain Heterogeneity, Immunomodulation, and Inflammation on the Tumour Microenvironment and Ovarian Cancer Progression

**DOI:** 10.3390/biom15121685

**Published:** 2025-12-02

**Authors:** Chrissie Giatrakis, Apriliana E. R. Kartikasari, Thomas A. Angelovich, Katie L. Flanagan, Melissa J. Churchill, Clare L. Scott, Srinivasa Reddy Telukutla, Magdalena Plebanski

**Affiliations:** 1 Accelerator for Translational Research in Clinical Trials (ATRACT) Centre, School of Health and Biomedical Sciences, STEM College, RMIT University, Melbourne, VIC 3083, Australia; chrissie.giatraki@gmail.com (C.G.); april.kartikasari@rmit.edu.au (A.E.R.K.); thomas.angelovich@rmit.edu.au (T.A.A.); melissa.churchill@rmit.edu.au (M.J.C.); 2 Centre for Infectious Diseases and Microbiology, Westmead Hospital, Sydney, NSW 2145, Australia; katie.flanagan@health.nsw.gov.au; 3 Clinical Discovery Translation Division, Walter and Eliza Hall Institute of Medical Research, Melbourne, VIC 3050, Australia; scottc@wehi.edu.au

**Keywords:** human cytomegalovirus, ovarian cancer, cancer progression, immunomodulation, inflammation, tumour microenvironment, viral strain heterogeneity, oncovirus, immunosuppression

## Abstract

The complex relationship between human cytomegalovirus (HCMV) and cancer has been of interest since the 1960s. As a highly prevalent human β-herpesvirus, HCMV establishes lifelong latency in CD34+ myeloid progenitor cells and has been implicated as an oncomodulatory virus in various cancers, including glioblastoma multiforme, breast, prostate, colorectal, and ovarian cancer (OC). Recently, discussions have emerged regarding the classification of HCMV as an eighth oncovirus due to the persistence of its nucleic acids and proteins in many tumour types. As one of the deadliest gynaecological cancers, OC is often characterised as the ‘silent killer’ with less than half of women surviving for 5 years, a rate that drops below 20% when detected at advanced stages. Reported effects of HCMV vary between cancers, likely due to differences in tumour type, viral strain, and disease stage. While HCMV infection has been linked to poor OC patient outcomes, its impact on the OC tumour microenvironment (TME) and immune system remains less understood. Investigating HCMV’s potential oncogenic role could provide critical insights into OC progression. This review discusses recent developments on HCMV’s multifaceted roles in OC, including strain heterogeneity, immunomodulation of the TME, dysregulation of inflammatory signalling pathways, and potential therapeutic approaches targeting HCMV in anti-cancer immunotherapies.

## 1. Introduction

### 1.1. Ovarian Cancer (OC)

Ovarian cancer (OC) is one of the deadliest gynaecological cancers, often referred to as the ‘silent killer’ due to its asymptomatic progression and frequent late-stage diagnosis [1,2]. Approximately 75% of women with OC are diagnosed at Stage III or IV, with their five-year survival rate being less than 30% [1,3,4]. Despite advances in surgery and chemotherapy, long-term survival remains poor, with many patients developing treatment resistance [1,2,5]. A major barrier to improving patient outcomes is the lack of reliable early diagnostic biomarkers and effective immunotherapeutic strategies [6]. Increasing evidence suggests that the OC tumour microenvironment (TME), particularly the immune landscape, plays a crucial role in disease progression and in response to treatment [6,7,8].

### 1.2. Human Cytomegalovirus (HCMV)

Human cytomegalovirus (HCMV) is a persistent β-herpesvirus that establishes lifelong chronic latency in host CD34+ myeloid progenitor cells [1]. While infection is typically asymptomatic in healthy immunocompetent individuals, it can cause severe disease in older adults and immunocompromised patients, such as transplant recipients and cancer patients [1,4]. Periodic reactivation of latent HCMV is well-documented and can be triggered by inflammation, stress, or immunosuppression [1]. The virus can infect a wide range of cell types, including epithelial cells, fibroblasts, monocytes, neural, and endothelial cells [7]. The association between HCMV and cancer has prompted increasing interest in the mechanisms by which it may contribute to tumour biology. Viral HCMV genes and proteins have been detected in a number of solid tumours such as glioblastoma multiforme (GBM) [9,10,11], breast (BC) [12,13,14,15,16], prostate [17,18,19], colorectal [14,20], and OC [1,4,21,22], where they have been proposed to potentially play a role in oncomodulation by promoting inflammation, angiogenesis, immune evasion, and tumour progression [1,7,23,24].

### 1.3. Prevalence of HCMV in Ovarian Cancer

In OC specifically, the current literature predominantly focuses on the impact of HCMV infection on overall survival (OS) outcomes and prognosis. These studies have identified the presence of viral HCMV proteins in OC and have correlated their expression to poor prognostic outcomes [6,21,25,26,27,28]. A study by Rådestad et al. [6] found that a higher grade of HCMV infection is correlated with advanced disease stage and worse OS in OC. Additionally, coinfection of HCMV with human papillomavirus (HPV), and Epstein–Barr virus (EBV) has recently been investigated [4,29]. Grabarek et al. [4] demonstrated a significantly higher risk of HCMV and HPV coinfection in patients with OC who underwent surgery, whereas patients who underwent both surgery and chemotherapy had a significantly higher risk of HPV and EBV coinfection. Similarly, Paradowska et al. [29] reported coinfection of HCMV and HPV in two-thirds of OC patients, suggesting that these viruses may play complementary roles in the development and/or progression of OC.

El Baba et al. [22] have also recently published multiple studies investigating how HCMV infection could drive cellular transformation, stemness, and tumour-like phenotypes in epithelial cells, particularly focusing on the induction of polyploidy giant cancer cells (PGCCs) and epigenetic reprogramming [7,11,13,16,21,22,30,31,32]. They demonstrated that HCMV infection promotes the formation of pro-oncogenic PGCCs, which other studies have shown are frequently detected in GBM, BC, and OC [9,11,13,16,17,21,22,30,31,32]. However, it is worth noting that the direct association between HCMV and PGCC induction in OC has yet to be established. The promising findings of these studies are paving the way for a renewed interest in this area of research, capable of potentially revealing new insights into the pathophysiology of ovarian tumours and deepening our understanding of HCMV’s involvement in OC.

Despite these intriguing findings, significant knowledge gaps remain in our understanding of HCMV’s role in OC progression. While associations with prognosis have been established, there is a notable lack of mechanistic studies investigating how HCMV directly influences OC cell behaviour, progression, and therapeutic resistance. Furthermore, comprehensive in vitro and tissue-based analyses examining HCMV’s molecular mechanisms in OC are limited. Thus, this review aims to discuss current knowledge on HCMV’s role in OC progression by examining recent findings from both OC research, and where OC-specific data is limited, drawing insights from HCMV studies in other cancer types. This review explores potential mechanisms by which HCMV may contribute to OC development and progression, synthesising evidence from related malignancies to propose how these mechanisms might apply in the OC context. Therefore, we seek to identify areas for future mechanistic studies and highlight the potential therapeutic implications of targeting HCMV in OC.

## 2. HCMV Viral Strain Heterogeneity and Oncogenic Potential in Ovarian Cancer

**Figure 1:**
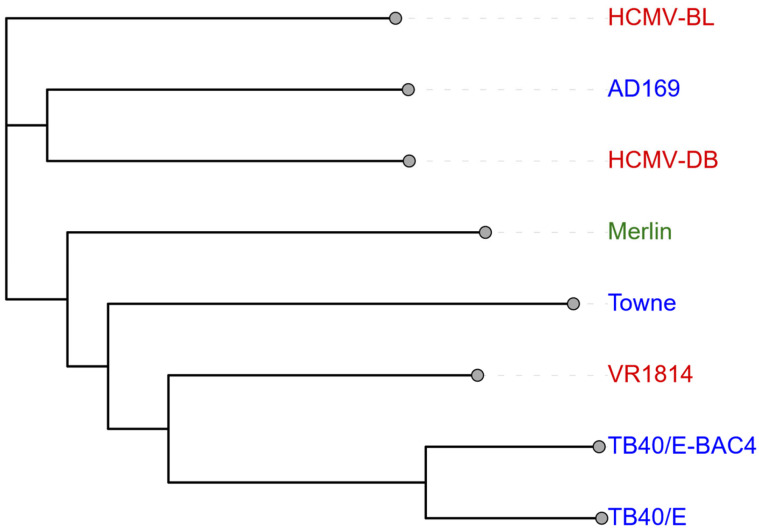
Comparison of the genomic sequences of common human cytomegalovirus (HCMV) clinical isolate and laboratory-adapted strains. Red indicates the clinical isolates, and blue indicates laboratory strains. Green indicates the known ‘prototype’ strain which although is classified as a clinical isolate, is highly passaged like laboratory strains. Sequences were obtained from the NCBI Nucleotide database. Accession numbers for the sequences used in this alignment are as follows: MW980585, FJ527563, KT959235, NC006273, FJ616285, GU179289, EF999921, KF297339.

HCMV is a very complex herpesvirus, displaying both intra- and inter-host variability [33,34]. Therefore, it is imperative that each viral strain is characterised and evaluated for its unique properties, as these variations may lead to significantly different research outcomes and biological effects. Viral HCMV strain heterogeneity plays a significant role in cancer progression. HCMV strains can be categorised as clinical isolates or laboratory-isolated. Common strains include HCMV-DB and HCMV-BL (clinical isolates), and HCMV-TB40/E (laboratory-isolated) [16,35]. In the context of known human viruses, HCMV has the largest genome (~236 kbp), with variabilities existing as mutations between strains (Figure 1) [33].

Ahmad et al. [16] also coined the terms ‘high-risk’ and ‘low-risk’ to describe the transformative ability of an HCMV strain. They reported that high-risk strains promote molecular oncogenic pathways (via the upregulation of key oncogenic proteins such as signal transducer and activator of transcription 3 (STAT3), phosphorylated retinoblastoma protein (pRB), and protein kinase B (Akt), etc.), demonstrate anchorage-independent growth (ability to form colonies in soft agar), and cause tumorigenicity in mice [16]. These strains are associated with the induction of pro-oncogenic signatures, development of polyploid giant cancer cells (PGCCs), overexpression of enhancer of zeste homologue 2 (EZH2), and enhanced epithelial-to-mesenchymal (EMT) plasticity [13,16,32]. Many studies have investigated the association between HCMV and the Myc/EZH2 axis, a critical pathway in tumorigenesis. These findings have consistently shown that high-risk strains (namely HCMV-DB and HCMV-BL) manipulate this pathway, thus supporting the potential role of HCMV as an oncovirus [11,16,17,21,31,32].

The *Myc* oncogene has been associated with tumorigenesis and poor prognosis in many cancers due to its ability to modulate key immune responses and suppress immunosurveillance [7,31]. It is also a regulator of EZH2 expression [31,32]. Overexpression of the epigenetic regulator EZH2 has been linked to tumour initiation, metastasis, and poor prognostic outcomes through its expansion of pro-tumour cells [32]. El Baba et al. [21] reported that use of EZH2 inhibitors in human ovarian epithelial cells can reduce HCMV replication, cell proliferation, and frequency of PGCC, further highlighting the important role of EZH2. Present in 37% of human tumours, PGCCs are linked to treatment resistance, metastasis and aggressive cancer phenotypes [13,17,32]. Recent studies have linked high-risk HCMV infection to the upregulation of PGCCs, EZH2, and the induction of EMT plasticity in various cancers including ovarian cancer [9,11,13,17,21,32]. The EMT is an important mechanism in cancer metastasis that enables cancer cells to detach from the primary tumour and become migratory and invasive [36]. Interestingly, this contradicts the findings of Oberstein and Shenk [37], who reported that HCMV infection induces a mesenchymal-to-epithelial (MET) transition in breast carcinoma and glioma stem cells. This discrepancy may be explained by their use of the laboratory-isolated HCMV-TB40/E strain and may suggest unique roles of strain heterogeneity on EMT/MET plasticity (Table 1) [37]. Recent research has implicated high-risk HCMV infection in PGCC development, and EZH2 overexpression in 72% of OC cases, supporting HCMV’s role in OC tumour progression [21].

### Research Limitations Due to Strain Variability

While these strain-specific effects demonstrate HCMV’s varied oncogenic potential, several limitations need to be addressed. High-throughput sequencing results reported by Patro [35] indicated significant genetic diversity between various HCMV clinical isolate strains, and even within a single host. These findings may explain the diverse oncogenic properties of viral strains [47]. By contrast, laboratory-based strains such as HCMV TB40/E are typically highly passaged in fibroblast cultures, thereby potentially altering their functional capacity [35]. The contradictions in the literature regarding EMT/MET transitions [11,37], and the paradoxical pro-inflammatory and immunosuppressive roles of HCMV, may be due to differences in tumour types, viral strains, or disease progression [48]. These differences may also act as confounding variables as to why there remain many unknowns surrounding HCMV pathology, and specifically in the context of cancer research. There are also practical considerations to note between both clinical isolates and laboratory strains. For the former, the virus is usually transferred via a cell-associated route, whereas the latter strains are typically cell-free. These routes differ in how the virus delivers its genetic material. Cell-associated delivery, also known as cell-to-cell delivery, involves viral transmission directly between an infected cell and a neighbouring cell through cell-to-cell contact [49,50]. However, in cell-free delivery, neighbouring cells are infected via viral particles which are released into the extracellular space by an infected cell [50]. Cell-associated viruses can more accurately represent in vivo conditions at the cost of longer cultivation times, whereas cell-free viruses are more time-efficient to work with, presenting them as an attractive experimental preference despite their potentially altered properties [35]. Consequently, these observations highlight the need for standardised and reproducible research protocols that systematically examine the oncogenic impacts of the same HCMV strains in different cancer types. This would set up a consistent reference framework allowing for comparison of strain-specific effects across studies—which is currently lacking in the literature. Additionally, comprehensive characterisation of known HCMV strains (such as their genomic, molecular, and genetic profiles) is needed to establish a reference library of strain properties. If the differences are considerable, this characterisation would assist future researchers in selecting the most appropriate strain for their specific research objectives.

## 3. Overview of HCMV Genes and Proteins in Immunomodulation and Oncogenesis

HCMV has been proposed to act as an oncomodulatory virus due to the presence of its viral gene products in various cancers, and the ability to modulate pro-oncogenic pathways, as summarised in Table 2 [23,51,52]. The key viral proteins, which have been detected in 90–100% of colorectal, breast, prostate, and neural-derived cancers, include HCMV 65 kDa tegument protein (pp65), and immediate early proteins 1 and 2 (IE1 and IE2) [14,51,53]. The viral-encoded pp65 is the most abundant HCMV protein and is associated with increased genomic mutations and immune evasion [7,52,54]. The virus also encodes for *US28*, which is a G protein-coupled receptor (GPCR) homologue that is thought to be crucial to establish latency; however, it is also expressed during productive infection [55,56]. Beisser et al. [56] identified *US28* as a key mediator of HCMV pathogenesis due to its constitutive activation of nuclear factor-κB (NF-κB) during lytic infection, ability to bind diverse chemokines, and potential role in immune evasion. The various HCMV gene products are expressed as a temporal cascade; therefore, their expression can indicate what stage of infection the virus has achieved [12]. As their name denotes, the two immediate early proteins, IE1 and IE2, can be used to denote that viral entry has occurred. These proteins are thought to affect various pathways to favour pro-oncogenic outcomes such as increased cell proliferation, tumour survival, and inflammation [7,24,53]. For early and late stages of infection, the expression of the *UL54* and *UL111A* genes are typically analysed, where the former encodes for viral DNA polymerase, and the latter for the cytomegalovirus-encoded human interleukin-10 homologue (cmvIL-10) [12]. There is another late-stage gene, *UL97*, which is involved in the reactivation of the virus from latency, and release of viral progeny [53]. Following primary infection, there is a robust adaptive immune response (both humoral and cellular) which is essential to create anti-viral HCMV antibodies before the virus enters the latency state [7]. This response is targeted against multiple viral products, but mostly specifically against the HCMV envelope glycoproteins B (gB) and H (gH) [7]. These two structural proteins are involved in tumour adhesion, invasion, and fusion of the viral envelope to the host cell membrane [7].

**Figure 2:**
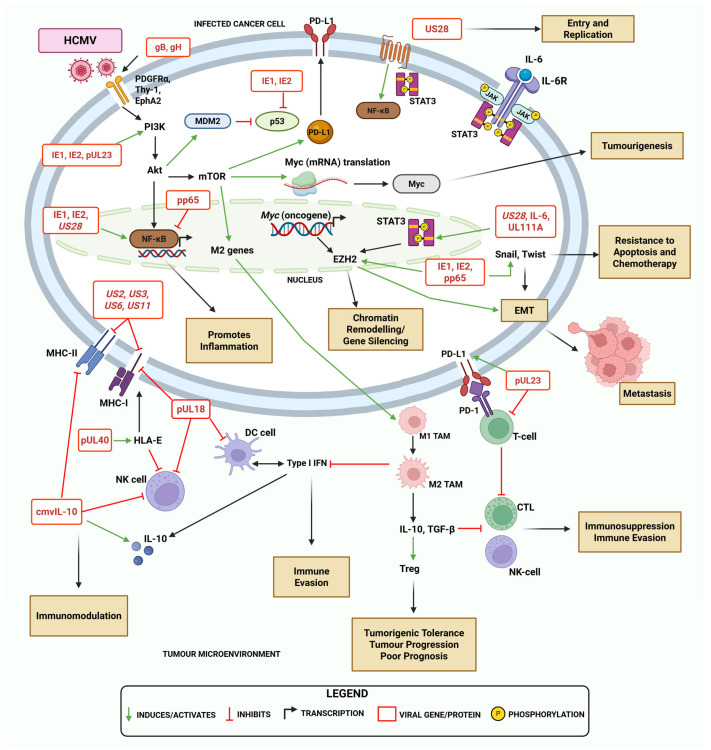
Proposed overview of HCMV-mediated modulation of the immune landscape to promote ovarian cancer (OC) progression. It is hypothesised that HCMV infection activates multiple interconnected pathways that can promote tumour growth, survival, and immune evasion in OC. Upon viral entry, HCMV proteins can activate the PI3K/Akt/mTOR signalling cascade, leading to enhanced cell survival and proliferation. This promotes programmed death ligand 1 (PD-L1) expression and immunosuppressive cytokine production (such as transforming growth factor-beta, TGF-β, and interleukin-10, IL-10). HCMV also promotes epigenetic reprogramming via the Myc/enhancer of zeste homologue 2 (EZH2) axis via the activation of mTOR [7]. This axis promotes the silencing of tumour-suppressor genes and loss of differentiation programmes, contributing to oncogenesis and EMT in OC [21,31]. The viral G protein-coupled receptor US28 constitutively activates NF-κB signalling and stimulates interleukin-6 (IL-6) production, which activates the JAK/STAT3 pathway via the IL-6 receptor (IL-6R) [7]. HCMV infection may also remodel the OC immune tumour microenvironment (TME) by promoting M1-to-M2 tumour-associated macrophage (TAM) polarisation, inhibiting T-cell, cytotoxic T lymphocytes (CTLs) and natural killer (NK) cell function through multiple mechanisms (pUL18, cmvIL-10 (viral Il-10 homologue), and human leukocyte antigen-E (HLA-E) upregulation), and downregulating class I and II major histocompatibility complex (MHC-I and MHC-II) presentation. Collectively, HCMV may exploit pro-inflammatory and immunosuppressive signalling to create a permissive environment for tumour progression, metastasis, and treatment resistance in OC. Adapted from sources [7,35]. Created in https://BioRender.com.

Primary HCMV infection alters the cellular secretome by inducing immunosuppressive mediators such as transforming growth factor-beta (TGF-β) and cmvIL-10, inhibiting effector T-cell and natural killer (NK) cell function while stimulating tumour-associated macrophages (TAMs), and inhibiting dendritic cell (DC) maturation [1] (Figure 2). Recent studies have established a relationship between HCMV in OC. Shanmughapriya et al. [26] and Carlson et al. [25] detected HCMV proteins expressed at different levels in ovarian tumour specimens, establishing that the virus is present in both the tumour and surrounding cells of the cancer. Studies have also identified an association between the presence of viral proteins (pp65 and IE) in ovarian tissue with poor survival outcomes (OS) in OC [6,25,27], suggesting that HCMV affects OC prognosis. Rådestad et al. [6] reported a significant association between extensive pp65 expression and a shorter OS. However, they also found that OC patients with a high immune response against HCMV (characterised as high HCMV-IgG levels) had a better prognosis. Further, Ahmad et al. [16] showed that IE1 and IE2 are key transcription factors involved in the induction of the pro-tumorigenic Myc/EZH2 pathway. They describe a ‘hit-and-run’ mechanism [57], which proposes that the IE proteins transform healthy ovarian cells into malignant cells, facilitating a potential link between viral infection and cancer progression [16,58]. The prevalence of HCMV in OC, and its observed impacts on patient prognosis, supports the idea that HCMV plays an oncomodulatory role in disease progression by potentially contributing to immune suppression and inflammation.

**Table 2:** Summary of HCMV genes and proteins implicated in oncogenesis and immunomodulation.

Gene	Protein	Function/Role	Stage of Infection
*UL123*, *UL122*	IE1, IE2	Transcription factors—activate Myc/EZH2, drive inflammation, cell proliferation, genomic instability, apoptosis, and immune evasion [7,12,16,35,56]	Immediate Early
*UL83*	pp65	Tegument protein—sequesters IE1, promotes immune evasion, inflammation, proliferation, and genomic instability [6,51,56]	Structural
*US28*	US28	Viral GPCR—activates NF-κB, binds chemokines, enhances immune evasion, tumour growth, cell survival [32,55,59,60]	Early/late *
*UL54*	pUL54	Viral DNA polymerase—essential for viral replication [12]	Early
*UL111A*	cmvIL-10	IL-10 mimic—suppresses MHC-II, T/NK cell function, enhances immunosuppression, migration, and metastasis [7,12,61,62]	Late *
*UL97*	pUL97	Viral kinase—promotes reactivation, progeny release, and immune evasion [56]	Late
*UL55*	gB, gH	Envelope glycoproteins—facilitate viral adhesion, entry, and fusion [7]	Structural
*UL40*	pUL40	MHC-I signal mimic—stabilises MHC (HLA-E) to inhibit NK cells via CD94/NKG2A receptor, supports tumour survival and immunosuppressive TME [1,7,63]	Late *
*UL18*	pUL18	MHC-I homologue—binds LIR-1 to inhibit NK cells, downregulates MHC-II, impairs dendritic cell development, and suppresses T-cell responses [1,7,63,64]	Late
*UL138*	pUL138	Latency-associated protein—establishes and maintains latency in vitro, supporting infected cell survival, and suppressing viral replication [61,62]	Latency
*LUNA*	LUNA	Latency Unique Natural Antigen (LUNA)—regulates viral reactivation, and latency-associated gene transcription [62,65,66]	Latency

* Denotes genes that are thought to be expressed during both phases of lytic (active), and latent infection.

## 4. HCMV-Mediated Mechanisms of Immune Modulation and Evasion in Ovarian Cancer

Infection by HCMV within the OC TME could potentially promote immune evasion via diverse mechanisms. At present, there is a lack of studies investigating the immune relationship between HCMV and OC. We therefore hypothesise that one such mechanism may involve direct infection and modulation of cancer cell function. The virus may also be able to evade the immune system by acting on immunosuppressive pro-tumour cells such as myeloid-derived suppressor cells (MDSCs), TAMs, and regulatory T-cells (Tregs) [1,6]. At the molecular level, HCMV can avoid immune recognition through modulation of both classes of the major histocompatibility complex (MHC-I and MHC-II), and evasion of NK-cell-mediated cytotoxicity [1,63,67]. Additionally, the virus may also promote oncogenesis by altering inflammatory signalling pathways, creating a pro-inflammatory environment that favours tumour progression [15,20]. Conversely, the inflammatory nature of the OC TME itself may facilitate and provide favourable conditions for HCMV replication and latency establishment, potentially even promoting viral reactivation [68]. An overview of these processes is detailed in Figure 2. This complex bidirectional relationship is yet to be fully elucidated and poses the question of whether HCMV-driven inflammation contributes to cancer-associated immunosenescence in OC patients.

### 4.1. Viral Induction of Cancer Stemness and Stem Cell Expansion

#### 4.1.1. HCMV Infection on Thy-1 and PDGFRα in Cancer

Human herpesviruses such as HCMV are known to exploit stem cells (SCs) to establish and regulate latency and reactivation [7,69]. The key SC reservoir for HCMV latency is the haematopoietic stem and progenitor cells (HPCs) found in the bone marrow [69]. Two stem cell-associated surface markers, Thy-1 cell surface antigen (Thy-1, also referred to as CD90) and platelet-derived growth factor receptor alpha (PDGFRα), have been identified as mediators of HCMV entry and replication in host cells [7,70]. Li et al. [70] identified Thy-1 as a key entry mediator and proposed that it may form a complex with the HCMV envelope glycoproteins gB and gH to facilitate viral entry. However, whether this interaction is direct, and how Thy-1 may function during the early stages of infection, remains unclear [70]. Notably, Thy-1 is also expressed on various other HCMV-permissive cell types, indicating it may function as a broader entry mediator [70]. Thy-1 has also been proposed as a putative cancer stem cell (CSC) marker in hepatocellular carcinoma [71], GBM [72], and BC [73]. In BC, Lobba et al. [73] reported that Thy-1 overexpression correlated with increased metastasis and reduced OS. Similarly, Connor et al. [74] found that in OC, Thy-1 marks a subpopulation of CSCs, and its high expression was linked to poor prognosis, driven by its role in promoting CSC self-renewal and proliferation. Despite these findings, the functional role of Thy-1 in cancer remains complex. Multiple studies have associated its expression with poor prognostic outcomes in GBM, hepatoblastoma, acute myeloid leukaemia, and BC [73,75,76,77], while others have identified tumour-suppressive effects in ovarian adenocarcinoma, neuroblastoma, and nasopharyngeal carcinoma [78,79,80]. It is worth noting that these discrepancies may be due to differences in cell populations. For instance, Connor et al. [74] assessed Thy-1 expression in ovarian CSCs (OCSCs) and found that knockdown reduced stemness features and self-renewal. Conversely, Abeysinghe et al. [78] overexpressed Thy-1 in differentiated SKOV3 OC cells, where it acted as a tumour suppressor. This suggests that Thy-1 may have a dual role in OC progression depending on the cellular context.

Another receptor, PDGFRα, has been proposed as a mediator of HCMV entry, primarily through its interaction with the gB/gH complex [81,82,83]. While early studies suggested that PDGFRα is essential for viral entry, recent findings have shown that HCMV can infect cells in the absence of PDGFRα, indicating that its role is not strictly required [81,83,84]. This suggests that PDGFRα may contribute to post-entry signalling events that support infection, rather than serving as a universal entry receptor [85]. Notably, PDGFRα is also expressed on various stem and progenitor cell types such as mesenchymal SC and neural progenitors, where it can regulate proliferation and self-renewal. In cancer, PDGFRα expression has been identified in GBM and BC stem-like cells, and its activation has been associated with an increase in mesenchymal traits and enhanced tumorigenicity [83,86]. Supporting this, Yang et al. [83] demonstrated that overexpression of PDGFRα significantly enhanced HCMV infection in BC cells, suggesting a functional role for this receptor in facilitating viral entry. However, compared to BC, the virus did not rely on PDGFRα for entry in fibroblasts, and instead promoted inflammatory signalling pathways [83]. These findings highlight the potential tumour cell-specific differences in receptor usage and downstream effects of HCMV.

#### 4.1.2. Potential Dual Role of PDGFRα on Promoting Ovarian Tumour Aggressiveness in HCMV Infection

In OC, emerging evidence has proposed a role for PDGFRα in the maintenance and acquisition of stem-like phenotypes, which can promote tumour aggressiveness and treatment resistance [87]. Given HCMV’s ability to exploit stem-like cells as reservoirs for infection and latency, PDGFRα expression on CSC-like cells may serve a dual function. That is, HCMV may facilitate viral entry into permissive cells, while simultaneously enabling HCMV to promote tumour progression via sustaining or enhancing stemness. Recent findings from Franciosa et al. [88] demonstrated that PDGFRα inhibition in OCSCs resulted in a reduced stemness phenotype. They also found that despite patient heterogeneity, the stemness molecular pathways of the OCSCs remain conserved and similar between different patients, highlighting the potential for targeted OCSC therapies [88]. However, Szubert et al. [89] also reported a high expression of PDGFRα in OC, but they did not find any correlation with prognosis or tumour stage. Given the established role of OCSCs in driving chemoresistance and recurrence [5], combining anti-OCSC therapies with anti-viral agents may offer a synergistic approach to limit both tumour progression and HCMV-driven oncogenic effects.

Recently, ephrin receptor A2 (EphA2) has also been implicated as a functional entry receptor for HCMV in GBM, where it mediates membrane fusion via interactions with the gH/gL complex [90,91]. Dong et al. [91] reported that overexpression of EphA2 in GBM cells was associated with poor patient prognosis. Notably, EphA2 has also been linked to proliferation, dedifferentiation, adhesion, and metastasis in OC [92], suggesting that it may play a similar role in this context. However, this has not yet been explored experimentally and warrants further investigation into EphA2 as a potential mediator of HCMV entry and a therapeutic target in OC.

#### 4.1.3. HCMV May Induce Tumorigenic Properties and Stemness Pathways in OC

HCMV infection has been shown to enhance cancer cell stemness and EMT, both of which are critical drivers of tumour progression and metastasis [18]. Studies in GBM [84], BC [30], and colorectal cancer [18] have reported that HCMV-IE proteins can upregulate stem cell markers, activate EMT transcription factors such as Snail and Twist, and promote resistance to apoptosis and chemotherapy. One mechanism through which HCMV may induce these effects is by causing cancer cells to acquire ‘stem-like’ properties, thereby generating cancer stem-like cells (SLCs) [18]. Interestingly, Teo et al. [18] were the first to demonstrate that HCMV preferentially infected colorectal SLCs rather than permissive fibroblasts, indicating that cancer SLCs may be a more favourable target for infection. The authors proposed that this increased susceptibility was due to the acquisition of stemness-associated features which allowed them to possess fibroblast-like characteristics [18]. This connection has yet to be established in OC; however, recent experimental evidence by El Baba et al. [22] has demonstrated HCMV’s ability to induce stemness features in ovarian epithelial cells. This group has published a series of studies highlighting the transformative capacity of HCMV in healthy ovarian cells [7,21,22,31,93]. Their most recent findings implicated HCMV-IE1 as a key viral protein involved in transforming normal ovarian cells into SLCs [22]. While these studies were conducted in non-malignant cells, they provide mechanistic insights into how HCMV infection may contribute to the acquisition of tumorigenic properties, potentially including in the context of OC development or progression. These viral effects reflect the phenotypes associated with Thy-1 and PDGFRα expression on SCs, suggesting that HCMV may not only exploit CSCs for entry, but also harness their pro-oncogenic signalling potential. In OC, this dual interaction could enhance tumour growth, invasiveness, and immune evasion. Together, these findings support the hypothesis that HCMV may directly target OCSCs and promote disease progression.

### 4.2. Impacts of HCMV on Antigen Presentation and Immune Checkpoint Modulation

HCMV is known to evade the immune system via multiple mechanisms [1,53,94]. One of these is the downregulation of classical MHC-I and MHC-II molecules [1]. Certain HCMV gene products (such as *US2*, *US3*, *US6*, *US10*, and *US11*) cause the downregulation of MHC-I, which prevents CD8+ T-cells from recognising HCMV-specific peptides [1,94]. Consequently, the ability of CD8+ and CD4+ T-cells to recognise HCMV-infected cancer cells is impaired, facilitating immune evasion and persistent infection in tumours [1]. Importantly, MHC-I downregulation is a common feature of many cancer types, including OC, where it serves as a tumour-immune evasion mechanism [8]. The downregulation of MHC by HCMV has been studied in various cancers such as in GBM, where it acts as an attractive target for different immunotherapies [7,53,95]. In addition to MHC modulation, HCMV has developed strategies to evade NK-cell-mediated cytolysis [1,53]. The HCMV-encoded protein pUL18 (an MHC-I mimic) competitively binds to inhibitory NK cell receptors, and pUL16 inhibits the expression of NK-cell-activating ligands, resulting in an inhibition of NK cell action [53]. Most OC tumours are considered ‘cold’ tumours (as opposed to hot or inflamed), because of their low presence of tumour-infiltrating lymphocytes (TILs), impaired T-cell activity, immunosuppressive TME, and poor response to immunotherapy [96,97]. Given that OC already has limited antigen presentation, HCMV infection may worsen this immune evasive state by further suppressing MHC-I expression and compounding the existing immune dysfunction.

#### 4.2.1. Upregulation of PD-L1 by HCMV in Cancer

Additionally, Yuan et al. [94] demonstrated that the *UL23* gene provides another way for HCMV to evade T-cell-mediated cytotoxicity. Besides the downregulation of MHC-I molecules on infected cells, the study was able to demonstrate that *UL23* upregulates the expression of programmed death ligand-1 (PD-L1) [94]. This proposes a new mechanism for how HCMV can evade T-cell activity, especially as most studies at present mainly focus on the effect of the MHC downregulation on T-cells. While the roles of PD-L1 in a tumour context have been extensively studied, little is known about its interactions with HCMV infection and immune evasion [94]. Infection with HCMV has been shown to upregulate PD-L1 levels in cancers such as melanoma, gastric cancer, and GBM [98,99,100]. Similar observations have been reported for EBV and HPV infections in cancer [101,102]. Increased expression of PD-L1 in cancer cells and in the TME increases the ability of tumours to escape immune surveillance detection [99]. These findings are corroborated by Feng et al. [98] who implicated that the *UL23*-encoded protein (pUL23) causes the overexpression of PD-L1 via the PI3-Akt signalling pathway. They also demonstrated a correlation between this overexpression with reduced sensitivity of tumour cells to T-cell-mediated cytotoxicity and enhanced T-cell apoptosis. Thus, HCMV was shown to weaken anti-tumour immunity via the actions of pUL23 on PD-L1 upregulation in gastric cancer, and the inhibition of CD8+ T-cell infiltration, reducing the expression of inflammatory markers in the TME [98]. Similar findings were reported by Qin et al. [100], who showed an increase in PD-L1 expression in GBM following HCMV infection, and that this occurs only in glioma cells as opposed to normal surrounding cells. Multiple studies have shown that PD-L1 expression is significantly correlated with shorter OS [100,103,104,105,106], thus supporting another way HCMV may be able to promote tumour progression and develop a more aggressive cancer phenotype. It is worth noting that the prognostic effect of PD-L1 may vary based on which cell it is expressed on [107,108]. The *BRCA1/2*-mutated high-grade serous OC (HGSOCs) subtype is typically characterised as a ‘hot tumour’, due to its high expression of PD-L1+ TILs, where PD-L1 expression is correlated with improved prognostic outcomes in an immunotherapy context [107]. The nature of PD-L1 expression as a predictive biomarker for positive responses to immunotherapy in OC remains controversial. Some studies associate high PD-L1 expression with poorer OS [109,110,111], whereas others suggest it may be a positive factor for progression-free survival (PFS) and OS [112,113]. Promising findings from Li et al. [108] utilise spatial analysis in OC to further define the known relationship between PD-L1 and OC. Following on the work of previous studies that correlated high PD-L1 expression on macrophages with improved survival in OC, Li et al. [108] found that the location of these PD-L1+ macrophages in the TME can predict poor prognostic outcomes. They reported that PD-L1+ macrophages found within 30 μm of CD8+ T-cells in the OC TME are significantly correlated with poor OS and poor disease-free survival (DFS) [108]. Therefore, PD-L1 appears to have a potential dual role, where its expression on immune cells appears to improve patient outcomes, as compared to when it is expressed on cancer. Given HCMV’s demonstrated capacity to upregulate PD-L1 in other cancer types, these findings highlight the need to determine which OC cells express PD-L1, where they are located in the TME, whether it is modulated by HCMV, as well as to consider how different therapeutic approaches may interact with these factors to affect OC prognosis. 

#### 4.2.2. HCMV-Induced Reprogramming of Macrophages Towards a Pro-Tumour Phenotype in the OC TME

Beyond lymphocyte modulation, HCMV may also further suppress immunity in the TME by the reprogramming of TAMs towards an M2 tumour-promoting phenotype [1]. This reprogramming is driven by viral mechanisms that cause the M2 TAMs to secrete pro-inflammatory cytokines (such as IL-10 and TGF-β), activate STAT3 signalling, and suppress type I interferon responses [1,53]. Therefore, in cancer, it is hypothesised that HCMV may use the reprogramming of TAMs to drive migration and a pro-tumour environment, further contributing to immune evasion [53]. In GBM, cmvIL-10 has been proposed to promote PD-L1 expression which inhibits the actions of effector T-cells and NK cells, as well as contributes to the polarisation of TAMs into the M2 phenotype [1,114], though the mechanistic details in the tumour context require further validation. It is not known if a similar mechanism would operate in OC. Notably, Stevenson et al. [115] utilised global transcriptome analyses to propose that HCMV-infected TAMs are mostly polarised into an M1-like pro-inflammatory phenotype, while also upregulating M2-associated genes to dampen the immune response. This dominant TAM profile mirrors that of the one proposed in OC. A characteristic of the ‘cold’ immunosuppressive nature of ovarian tumours (in addition to low TILs), is the large number of TAMs and MDSCs [116]. Given that OC is characterised by an immunosuppressive, myeloid-rich TME, it is plausible that HCMV could exploit and amplify this phenotype, further enabling tumour progression and immune evasion. Whether HCMV infection modulates TAM phenotypes specifically within the OC TME remains an important question for future validation.

#### 4.2.3. HCMV Exploits the Human Leukocyte Antigen-E (HLA-E)/NKG2A Immune Checkpoint Axis to Evade T-Cell-Mediated Killing

As previously described, the classical MHC molecules are a key target of immunomodulation by both HCMV and cancers. Human leukocyte antigen-E (HLA-E) is a low polymorphic and conserved MHC-Ib molecule in humans [117]. Unlike its classical MHC counterpart, this molecule does not require binding to T-cell receptors (TCRs) [117]. HLA-E instead, acts independently to bind directly on the inhibitory NKG2A and activate NKG2C receptors, which are present on NK cells and some CD8+ T-cells [117,118]. The key peptides in the HLA-E signalling pathway are the VL9 signal peptide, and the peptide transporter associated with antigen processing (TAP) [117,119]. Generally, HLA-E has low cell surface expression, but certain pathogens and cancer cells can upregulate its expression [117]. Overexpression of HLA-E results in inhibition of NK-cell-mediated cytotoxicity via the NKG2A/CD94 receptor and provides a mechanism for viruses (such as HCMV) and cancers to evade immune clearance [1,100,117,119]. There are two proposed ways by which HCMV exploits HLA-E during infection [120]. Firstly, the downregulation of MHC-I helps to avoid immune recognition but also reduces binding competition to VL9 [117]. This increases the presence of VL9 peptides available for HLA-E. However, HCMV also blocks TAP signalling, reducing the stability of endogenous VL9. Secondly, Ulbrecht et al. [120] discovered that the viral *UL40* gene encodes for a TAP-independent ligand that mimics VL9, leading to the increased expression of HLA-E, despite MHC-I inhibition. The findings of Tomasec et al. [121] corroborated this, suggesting HCMV’s dual strategy of escaping both antigen presentation and NK lysis by exploiting the expression of classical (MHC-I) and non-classical (HLA-E) MHC molecules. While these HCMV-specific mechanisms involve viral proteins, tumours can also upregulate HLA-E expression through alternative pathways [117]. Overexpression of HLA-E has been linked to various solid tumours such as colorectal [122,123,124], breast [125], GBM [126], gastric [127], and gynaecological cancers [128,129]. In addition, the upregulation of HLA-E is often associated with increased NKG2A expression on CD8+ TILs and presents this receptor as an attractive target for immunotherapies [117]. Notably, HLA-E overexpression in cancers has also been associated with poor prognosis in GBM [126], gastric cancer [127], and multiple myeloma [130]. Intriguingly, Gooden et al. [128] reported that despite high expression of HLA-E in cervical cancer, there was no effect on prognosis. The complete role of HLA-E on OC is not yet fully understood, as reflected by inconsistent findings in the literature. Certain studies have stated that HLA-E expression alone may not serve as a reliable prognostic biomarker due to its ubiquitous expression on cells, and that it may act in combination with HLA-G (another non-classical MHC molecule) instead [127]. A study by de Kruijf et al. [125] found that a low MHC-I profile with high HLA-E and HLA-G correlated with reduced OS and increased metastatic potential in BC. Similarly, in colorectal cancer, Guo et al. [122] reported a significant correlation between HLA-E and HLA-G on prognosis. They found that HLA-G expression alone is a factor for OC, whereas HLA-E alone or in combination with HLA-G is not [122]. These findings contradict those of Zhen et al. [123] who observed a correlation between HLA-E overexpression and the poorest long-term OS in Stage III colorectal cancer. Therefore, the immunoregulatory effects of HLA-E and HLA-G overexpression on cancer prognosis require further investigation. This relationship is even more complex in OC. Gooden et al. [128] showed that the negative prognostic effect of HLA-E in OC is conferred by the low presence of cytotoxic T-lymphocytes (CTLs) in the ‘cold’ TME. This results in a positive feedback loop, where the high ratio of HLA-E effectively neutralises the benefits of CTL infiltration, furthering OC tumour resistance to NKG2A-mediated lysis [128]. By contrast, there are approximately three times more circulating CTLs in the cervical cancer TME, dampening the prognostic effect of HLA-E [128]. Notably, recent single-cell and proteogenomic analyses have surprisingly shown high expression of both MHC-I and II in epithelial OC, including presentation of tumour-associated peptides on MHC-II [8]. This challenges the pre-existing notion that tumours such as OC evade immune recognition primarily through MHC downregulation and instead suggests that OC may maintain antigen presentation and rely more heavily on immunosuppressive techniques (via HLA-E) as a key strategy of immune evasion. Given evidence of HCMV presence in OC [26,29], this raises the possibility that HCMV-mediated MHC downregulation in infected tumours may further contribute to immune escape by shifting the balance toward HLA-E-mediated NK cell inhibition, compounding the tumour’s existing immunosuppressive mechanisms. Given the critical role of the HLA-E/NKG2A axis in immune evasion by both HCMV and cancers, novel therapeutic approaches are being developed to target this pathway. Sætersmoen et al. [131] recently pioneered a chimeric ‘A/C switch’ receptor which targets cancer cells with high HLA-E expression to activate rather than inhibit the cytolytic functions of T and NK cells. This represents a highly promising anti-cancer strategy, which theoretically could also potentially be repurposed to target HCMV-infected cells that overexpress HLA-E, thereby redirecting immune responses toward viral clearance rather than inhibition. However, this application remains to be tested experimentally in OC. These dynamics highlight the importance of upstream immune signalling pathways that regulate MHC and HLA-E expression, including cytokines involved in anti-tumour responses.

#### 4.2.4. Interferon-γ Signalling as a Key Feedback Regulator in HCMV Infection and Cancer Progression

One such pro-inflammatory cytokine is interferon-γ (IFN-γ), which is secreted by activated T-cells, NK cells, and γδ T-cells and is involved in anti-viral and anti-tumour immunity [6,94,119,132]. However, in the context of HCMV infection and OC, IFN-γ signalling may paradoxically contribute to immune evasion. This is particularly relevant in HCMV-infected tumours, where IFN-γ-rich environments may establish a negative feedback loop where immune activation induces checkpoint molecules (such as PD-L1 and HLA-E), which in turn suppress immune effector function [94,109]. Studies by Yuan et al. [94,133] have demonstrated that the HCMV *UL23*-encoded protein, pUL23, inhibits IFN-γ signalling via the inhibition of the STAT1 pathway, allowing HCMV to evade T-cell-mediated lysis. This study addressed gaps in the literature surrounding the full role of HCMV’s inhibitory effect on T-cell responses and proposed pUL23 as a key immunoregulator of IFN-γ signalling and immune escape [94]. Additionally, they were the first to report that HCMV may promote immune evasion by using PD-L1 as an intercellular inhibitory signal that evades CTL killing in co-culture assays of HCMV-infected cells with T-cells [94]. IFN-γ has also been shown to upregulate both PD-L1 and HLA-E expression in OC cells via the Janus-associated kinase (JAK)/STAT1 pathway, leading to the suppression of cytotoxic T-cell and NK cell activity [119]. This was also observed by Malmberg et al. [134], who reported overexpression of HLA-E and HLA-G in OC cell lines treated with IFN-γ, which conferred the cells from CTL-mediated lysis. Although PD-L1 and MHC-I are often co-regulated by IFN-γ in inflamed tumours, OC frequently shows high PD-L1 with low MHC-I expression [98,119,135], suggesting alternative regulatory mechanisms. Given HCMV’s demonstrated association of PD-L1 and MHC-I expression in other cancer types, and the presence of HCMV in OC tumours, viral modulation could be a plausible (however not yet demonstrated) driver of immune evasion in OC. HCMV infection also promotes persistent expansion of γδ T-cells with a terminally differentiated effector memory T-cell re-expressing CD45RA (TEMRA) phenotype that produces IFN-γ, further skewing the immune landscape [94,132]. While early IFN-γ production by γδ T-cells and NK cells can enhance anti-viral responses, sustained IFN-γ signalling may ultimately drive upregulation of HLA-E, impairing NK cell cytotoxicity and correlating with poorer PFS in OC patients [119]. These findings suggest that in the context of HCMV infection, IFN-γ may have a dual role, by initially triggering anti-viral defence but eventually fostering an immunosuppressive TME via HLA-E and PD-L1 upregulation. However, this specific population has not been characterised in the OC TME context. Thus, these findings suggest that in infected OC tumours, HCMV may further hijack the dysregulated signalling of IFN-γ pathways in the TME to amplify existing immune escape mechanisms, potentially contributing to OC progression. However, direct experimental validation of these interconnected mechanisms specifically within the OC context is needed to confirm this proposed model.

#### 4.2.5. The Duality of HCMV’s Potential Protective and Immunosuppressive Roles in Immune Checkpoint Blockade Therapy

Recently, Milotay et al. [99] demonstrated that in metastatic melanoma, HCMV seropositivity is associated with a more cytotoxic and clonally expanded CD8+ T-cell environment, which enhances responsiveness to immune checkpoint blockade (ICB). Their findings suggest that anti-PD-1 therapy is particularly effective in patients with a pre-existing TEMRA phenotype, and that HCMV infection may condition the immune system to respond more robustly by driving cytotoxic differentiation and effector function in CD8+ T-cells [99]. Intriguingly, this study proposed that HCMV may play a protective role in melanoma, as HCMV seropositivity was negatively associated with the likelihood of a melanoma diagnosis [99]. Interestingly, while HCMV appears to be often linked to immune evasion in multiple solid tumours, its role in OC remains incompletely understood. By contrast, the findings of the recent study in melanoma show that HCMV may enhance responsiveness to ICB. Therefore, these contrasting findings highlight the complexity of HCMV’s immunomodulatory effects, which may vary depending on the tumour type, mutational landscape, and the balance of immune cell subsets in the microenvironment—all of which are critical factors in OC progression.

### 4.3. Viral Promotion of Inflammation and Its Effects in the TME

#### 4.3.1. Viral Activation of NF-κB Drives Production of Inflammatory Cytokines

NF-κB is a master regulator in the innate immune response of a host to a pathogen, such as a virus, and can stimulate the secretion of various inflammatory mediators [60,136,137,138]. Many pathways modulated by HCMV can appear contradictory, as the virus may act on the same pathway to cause both immunosuppression and inflammation. As described in Section 3, NF-κB plays a role in immune evasion when downregulated by pp65; however, it can also promote inflammation. Inflammation is a key player in carcinogenesis and has been associated with the reactivation and replication of HCMV [20]. The HCMV envelope glycoproteins (gB and gH) can interact with toll-like receptors 2 (TLR2) to stimulate the NF-κB pathway, resulting in the production of pro-inflammatory cytokines [1,132], such as interleukin-6 (IL-6) and tumour necrosis factor-alpha (TNF-α) [132]. In addition, HCMV can activate NF-κB and the PI3K pathway to promote the increase in M2-type TAMs, as previously described [136]. The HCMV-encoded *US28* acts as an oncogene due to its constitutive activation of NF-κB signalling during active infection [53,60]. The *US28*-mediated activation of NF-κB has been shown to increase inflammatory mediators often associated with several cancers such as IL-6, cyclooxygenase-2 (COX-2), and the activation of the STAT3 pathway [60,136]. Thus, the dysregulated activation of NF-κB by pathogens such as HCMV can contribute to a more inflammatory TME which may be favourable for tumour growth and progression.

#### 4.3.2. Inflammatory Signalling Pathways in Ovarian Cancer and Their Modulation by HCMV

**Figure 3:**
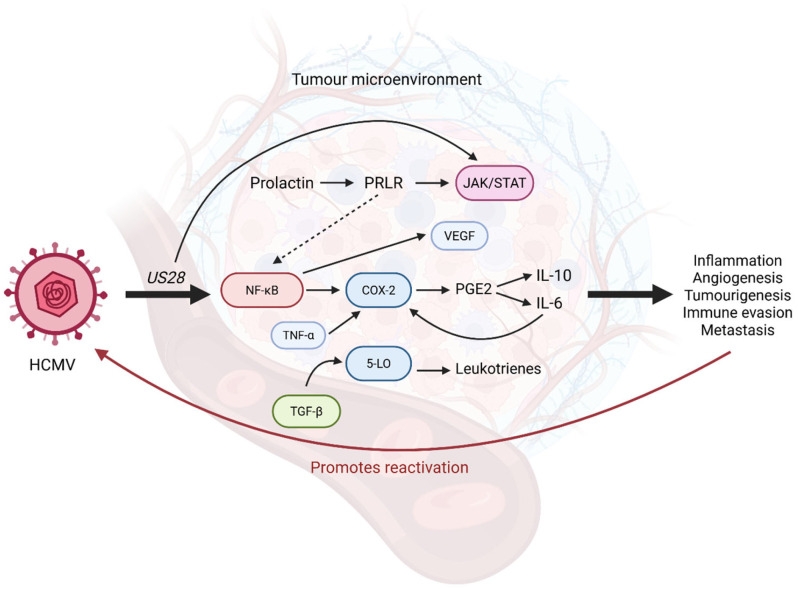
HCMV-mediated inflammatory signalling pathways to foster a pro-tumour microenvironment in cancer. During active infection, HCMV gene US28 constitutively activates NF-κB which is a regulator of pro-inflammatory enzymes such as cyclooxygenase-2 (COX-2) and 5-lipoxygenase (5-LO) [15,20,60]. Activation of COX-2 by NF-κB results in the production of prostaglandins such as PGE2 which can upregulate pro-inflammatory cytokines (such as interleukin-6; IL-6) as well as immunosuppressive cytokines (such as interleukin-10; IL-10) [15,20]. HCMV-mediated upregulation of COX-2 has been observed in various cancers [10,15,20,60,139]. 5-LO expression is also upregulated with HCMV infection, resulting in the metabolism of arachidonic acid to form leukotrienes which are implicated as pro-inflammatory mediators and contributors to cancer progression, immune evasion, and metastasis [15,20]. The upregulation of PGE2 and leukotrienes can attract inflammatory cells to the site of infection which promote inflammation of the tumour microenvironment (TME) [20]. The inflammatory TME may form favourable conditions for tumour progression as well as HCMV replication. In addition, COX-2 can be induced by inflammatory cytokines such as IL-6 and tumour necrosis factor-alpha (TNF-α) [20]. 5-LO is stimulated by transforming growth factor-beta (TGF-β), another pro-inflammatory mediator present in the TME [20]. These factors further drive inflammation in the TME which can promote reactivation and replication of HCMV. US28 also stimulates the activity of vascular endothelial growth factor (VEGF) via its activation of NF-κB, which can promote angiogenesis [60]. Further, HCMV can also promote inflammation via the prolactin/prolactin receptor (PRLR) axis [140,141,143,144]. Prolactin interacts with its receptor, PRLR, to promote activation of the Janus-associated kinase (JAK)/signal transducer and activator of transcription (STAT) pathway which is a key pathway involved in inflammatory signalling [144]. PRLR may also induce NF-κB which further drives inflammation [144]. Therefore, HCMV can directly promote inflammation in the TME via a number of pro-inflammatory mediators, which can also act in a positive feedback mechanism to further drive inflammation, tumour progression, and viral reactivation. Created in https://BioRender.com.

Infection with HCMV has been shown to enhance expression of key pro-inflammatory enzymes such as COX-2 and 5-lipoxygenase (5-LO) in the TME of BC and colorectal cancer (Figure 3) [15,20]. COX-2 drives the expression of prostaglandins and creates a pro-tumour environment via the upregulation of immunosuppressive cytokines such as interleukin-10 (IL-10), and pro-inflammatory cytokines (such as IL-6) [15,20,28]. As discussed above, HCMV *US28* activates NF-κB signalling, which directly upregulates the expression of COX-2, as observed by Maussang et al. [60]. The pro-inflammatory enzyme 5-LO metabolises arachidonic acid to generate eicosanoids, that can suppress immunosurveillance and promote cancer metastasis and chemoresistance [15,20]. Notably, Rahbar et al. [28] reported that patients presenting with pelvic inflammatory disease have an increased risk of borderline ovarian tumours (BOTs) due to HCMV protein and 5-LO overexpression [1]. In addition, COX-2 has been demonstrated to contribute to US28-mediated tumourigenesis [60,139]. Recent studies have demonstrated a correlation between HCMV infection and enhanced COX-2/5-LO expression in GBM [58], colorectal [20], and breast cancers [15]. Recently, Touma et al. [48] suggested that HCMV’s effects on COX-2 and 5-LO expression can cause a more malignant BC phenotype and poor prognosis. While COX-2 and 5-LO have been implicated in BOTs rather than ovarian carcinoma [28], the role of these inflammatory pathways in cancer progression warrants further investigation into HCMV’s potential effects on these enzymes in OC. Additionally, ovarian tumours exhibit high levels of prolactin receptor (PRLR) [140]. Infection by HCMV is known to interact with the prolactin (PRL)/PRLR axis to promote inflammation and potentially contribute to tumorigenesis [141,142]. Rahbar et al. [140] reported extensive expression of PRLR in HCMV-infected ovarian tumours and demonstrated that HCMV transfection in OC cells can stimulate PRL and PRLR. Activation of PRLR by PRL stimulates the JAK/STAT pathway, which is a key regulator of inflammatory responses. Additionally, PRLR activation may also induce NF-κB signalling, contributing to a pro-inflammatory feedback cycle. However, this relationship requires further investigation in the OC context as some studies suggest PRL may inhibit NF-κB [143] or act in a dual cytokine-dependent manner [144]. It is worth noting that these studies investigated PRL in COVID-19 [143] and arthritis [144], respectively, indicating that these discrepancies may also be dependent on the disease context. Thus, Rahbar et al. [140] proposed another potential HCMV-mediated mechanism of tumour progression via inflammatory signalling. Should future studies establish a causal link between HCMV infection and OC progression, investigating viral markers as potential indicators of inflammatory tumour phenotypes may be warranted, though clinical validation would be essential. Further, the impact of HCMV infection on COX-2, 5-LO, and PRLR expression suggests the use of inhibitors and modulators of these enzymes as potential attractive therapeutic options in the treatment of various cancers [15].

#### 4.3.3. HCMV-Induced Cytokine Dysregulation in Ovarian Cancer Ascites and Impact on the Tumour Microenvironment

**Figure 4:**
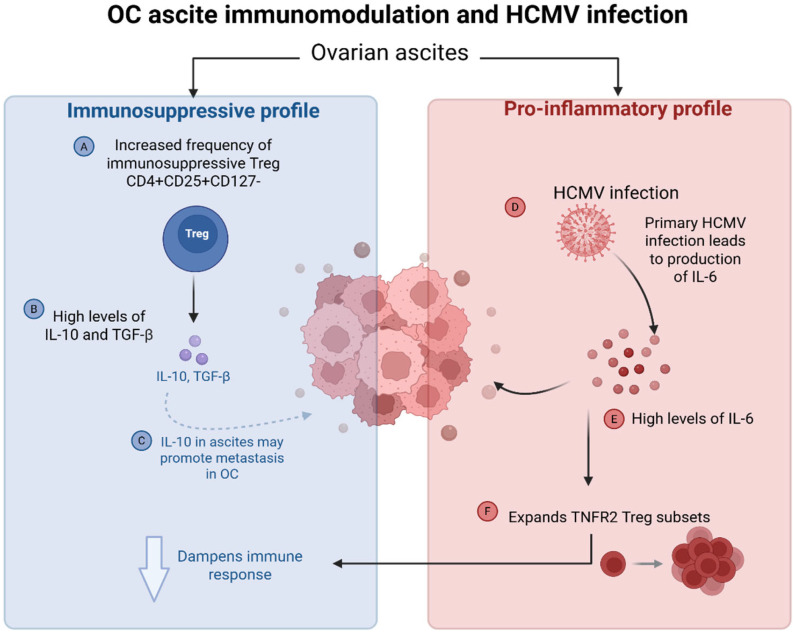
Immunomodulatory effects of ovarian cancer ascites on the tumour microenvironment and the role of HCMV infection. (A) There is an increased frequency of immunosuppressive regulatory T-cell (Treg) CD4 + CD25 + CD127- subsets in ascites [1,15,145,146]. (B) The fluid of ovarian ascites contains high levels of interleukin-10 (IL-10) and transforming growth factor-beta (TGF-β) which promotes an immunosuppressive profile [1,147]. (C) Findings from a preprint suggest that IL-10 in ascites may promote metastasis via the STAT3/ERK1/2 pathway [147]. (D) Infection with HCMV has been shown to increase the production of interleukin-6 (IL-6), (E) which has been found to act as an inflammatory mediator via its high levels present in ascites [1,145,146]. (F) The IL-6 cytokine also contributes to the immunosuppressive nature of ascites via the expansion of TNFR2+ Treg subsets which causes a dampened immune response [1,145]. This highlights the balance and interactions between pro-inflammatory and immunosuppressive components of ascites. Created in https://BioRender.com.

In their 2021 review, Cox et al. [1] suggested a potential link between ovarian ascites and HCMV-driven inflammation of the TME. Ascites, an accumulation of fluid in the peritoneal cavity, is found in a third of OC patients and contains both immunosuppressive cells and pro-inflammatory mediators [1,145]. While Tonetti et al. [146] characterised ascites as having an inflammatory profile due to the presence of high levels of the pro-inflammatory cytokine, IL-6, Cox et al. [1] emphasise their primarily immunosuppressive nature, with high levels of IL-10 and TGF-β. This contradiction reflects the dual role of IL-6—that is, while pro-inflammatory in nature, it can paradoxically promote immunosuppression through its expansion of Treg populations [145]. Active HCMV infection has been implicated in promoting an inflammatory profile in the TME through the increased production of IL-6 (Figure 4) [15]. Tonetti et al. [146] demonstrated an increased frequency of immunosuppressive Treg CD4 + CD25 + CD127- subsets in ascites, which is consistent with the 2021 review [1]. Based on findings by Kampan et al. [145], Cox et al. [1] suggest that IL-6 is a key player in immune modulation in OC ascites via its expansion of TNFR2+ Tregs subsets via STAT3, resulting in a dampened immune response. Interestingly, a preprint by Huang et al. [147] (2024) suggests that the presence of IL-10 in ovarian ascites can promote metastasis in OC via the STAT3 and extracellular signal-regulated kinase 1/2 (ERK1/2) pathways, though further validation through peer-reviewed studies is necessary to confirm these results. Transcriptomic analysis by Reinartz et al. [148] support these findings, as they demonstrated that the STAT3 signalling pathway is strongly activated in OC ascites. They report that both IL-6 and IL-10 were expressed on TAMs and tumour cells, and that their signalling is critical for disease progression and early relapse in OC patients [148]. These findings highlight the complex interplay between immunosuppressive and pro-inflammatory elements in the OC TME, demonstrating how HCMV can exploit and promote these oncogenic mechanisms to drive tumour progression.

## 5. Persistent HCMV Infection on Immunosenescence in OC Patients

The prolonged viral replication in HCMV infection has been shown to increase HCMV-specific memory T-cell inflation [7,99]. T-cell inflation refers to the phenomenon where HCMV-specific CD8+ T-cells progressively accumulate to extremely high frequencies over an individual’s lifetime due to repeated viral reactivation, rather than contracting after infection as typically occurs with other viruses [99]. Specifically, it is the induction of TEMRA inflation that is observed in HCMV infection [99]. This high fraction of HCMV-specific T-cells in healthy seropositive individuals allows for lifetime protection from the virus but not necessarily full clearance [7,99,149]. These factors, along with an inverted CD4:CD8 T-cell ratio, characterise the immune risk profile (IRP) observed in HCMV infection [1]. Further, this IRP has been associated with inflammaging and immunosenescence in the elderly [1,150]. It is also worth noting that these characteristics are observed during acute HCMV infection, as well as chronically infected cells. There may be a promising intersection between HCMV-induced immunosenescence and chemotherapy-induced immunosenescence in patients with cancer, which remains to be elucidated. With more than two-thirds of OC patients being aged 55 years or older [3]—and with a high prevalence of HCMV proteins being detected in OC specimens, we hypothesise that their immune systems may reflect that of natural ageing (immunosenescence) in the elderly. To our knowledge, no study has directly explored the possibility that HCMV exacerbates the immunosenescent profile in older patients with OC. We suggest that the virus may contribute to inflammaging and impaired immune surveillance in this population. Further investigations into the positive feedback relationship between HCMV, inflammation, immunosenescence, and thus inflammaging, may reveal new insights into the impact of the virus on the immune system and how this differs in cancer patients.

## 6. Therapeutic Implications

Despite increasing evidence that HCMV may contribute to OC progression, very few studies have explored therapeutic strategies that directly target HCMV in patients. Two promising immunotherapeutic approaches have emerged from studies in other cancers: T-cell-epitope-delivering antibodies (TEDbodies) and antibody–peptide epitope conjugates (APECs) [151,152,153]. Both methods redirect anti-HCMV CD8+ T-cells to cancer cells but differ in their mechanisms. TEDbodies insert viral antigens on MHC-I complexes for presentation [151], whereas APECs use cancer-associated matrix metalloprotease-cleavable linkers to release viral epitopes for presentation by endogenous MHC-I complexes [152,153]. Jung et al. [151] demonstrated that TEDbodies reduced tumour volume in breast and colorectal cancer xenografts, with enhanced efficacy when combined with anti-OX40 but not pembrolizumab. However, TEDbodies require functional antigen presentation pathways, which are often disrupted in cancer [151]. APECs have been developed to overcome this limitation and showed tumour volume reduction and improved survival in multiple cancer models [152,153]. For OC specifically, Zhang et al. [153] identified MMP7-HCMV APEC (EpCAM-MC) as a potent candidate that elicited robust anti-HCMV T-cell responses in patient-derived ascites and achieved a 50% reduction in tumour burden in xenografted mice. Both approaches require endogenous MHC-I complexes, which may be downregulated in certain cancers [154].

Another existing approach studied in various non-ovarian cancers, is the use of intra-tumoural (i.t) injection of anti-HCMV peptide epitopes, which is comprehensively described in the review by Britsch et al. [154]. These injections locally activate anti-HCMV T-cell responses by loading the virus-derived peptide epitopes that target MHC-I and MHC-II molecules expressed on cancer cells in the TME [154]. As opposed to TEDbodies and APECs, the i.t injection also delivers MHC-II restricted HCMV peptide epitopes which are presented on empty MHC-II molecules present on APCs, and promotes the anti-tumoural activity of both anti-HCMV CD8+ T-cells and CD4+ T-cells [154]. This was demonstrated by Çuburu et al. [155], who found that i.t injection of these peptide epitopes in mice was able to activate pre-existing anti-HCMV T-cell responses in mice xenografted with various tumour (lung, colon, and melanoma) models. Their findings exhibited strong anti-tumour effects via the expansion of HCMV-specific effector T-cells, activation of both adaptive and innate immune responses in the TME, and improvements in tumour control—ultimately conferring the mice with long-term anti-tumour immunity [155]. Although not yet investigated in OC, the demonstration of efficacy across multiple solid tumour types suggests this approach could warrant evaluation in OC models, particularly given the prevalence of HCMV in ovarian tumours.

There is also an increasing interest in the use of anti-HCMV T-cells in adoptive cell therapy (ACT) [154]. The goal is to expand the subset of anti-HCMV T-cells which can respond to and eliminate viral antigens by transducing chimeric antigen receptors (CARs) and TCRs into anti-HCMV CD8+ T-cells ex vivo [154]. Engineered CAR T-cells have been used in GBM patients with beneficial clinical responses. Given that both GBM and OC are characterised by immunosuppressive microenvironments and treatment resistance, evaluating similar approaches in OC may be appropriate, though OC-specific studies are needed to assess feasibility [154,156]. An advantage of using anti-HCMV T-cells in ACT is that they have long-term immune persistence due to memory T-cell inflation and lack of exhaustion, which overcomes a common issue faced due many cycles of ex vivo expansion of CAR T-cells [154,157]. This approach is currently being tested in clinical trials in a vaccine for the treatment of non-Hodgkins’s lymphoma [156]. There are also multiple vaccines currently under investigation that aim to induce anti-HCMV immunity [154,158,159,160].

In addition to immunotherapies, anti-viral drugs represent a promising therapeutic approach for targeting HCMV in cancer treatment. Current HCMV treatments include gangiclovir, valganciclovir, cidofovir, and foscarnet [161], though prolonged use can cause cytotoxic effects and therapeutic resistance, especially in the treatment of immunocompromised patients [19,154,161]. Baryawno et al. [162] demonstrated that gangiclovir (DNA polymerase inhibitor) reduced HCMV cell growth and replication in vivo, with enhanced efficacy when combined with celecoxib (COX-2 inhibitor). This combination was tested for cytotoxic effects in HCMV-negative cell lines (the study used MRC-5 fibroblasts), and they found that the same concentrations that inhibited tumour growth in medulloblastoma, had no effect on the viability of the fibroblasts [162]. These findings suggest an HCMV-specific effect of this combination treatment in medulloblastoma without affecting non-infected cells. Similarly, following up on the findings of their previously conducted retrospective analysis [163], Stragliotto et al. [164] reported promising findings using valganciclovir as an add-on treatment to GBM patients already receiving standard therapy. This combination treatment significantly improved both median OS and 2-year survival rate in GBM patients, compared to the control group (patients who only received standard of care treatment) [163,164]. In addition, they found that valganciclovir significantly improved OS following a recurrence of GBM, with the most significant improvement observed in patients who underwent surgery for tumour recurrence [163]. Importantly, valganciclovir doubled one-year survival and tripled two-year survival following recurrence, regardless of surgical intervention [163]. This is particularly relevant in the context of OC, which is often defined by high recurrence rates of approximately 25% and 80% in patients with early and advanced stages of disease, respectively [142]. Given the limited treatment options for recurrent OC and the favourable safety profile of valganciclovir demonstrated in GBM, evaluating this approach in recurrent OC represents a potential therapeutic strategy. A recent study by Classon et al. [19] evaluated multiple anti-HCMV drugs in prostate cancer models. Aciclovir and gangiclovir induced DNA damage and apoptosis during viral latency, while valaciclovir treatment in xenografted mice increased apoptosis, reduced tumour volume, and improved OS [19]. Novel agents (mithramycin A, maribavir) demonstrated superior efficacy compared to aciclovir, with earlier onset and greater anti-proliferative effects [19]. These effects were most pronounced in cells sensitive to IE1/2 knockdown, suggesting HCMV-dependent mechanisms [19]. If similar latent HCMV infection occurs in OC patients, these agents may have comparable therapeutic potential.

Ultimately, these findings also support the proposed concept of HCMV acting as an oncomodulatory virus, where its inhibition could potentially reduce cancer progression. Therefore, integration of HCMV-targeted therapies with standard OC treatments represents a promising clinical approach. Anti-viral drugs could be administered alongside platinum-based chemotherapy, while immunotherapeutic approaches (such as APECs and ACT) may be most effective as maintenance treatments when immunosuppression is reduced. Tailoring these therapies to HCMV-positive tumours could help identify patient subgroups most likely to benefit, especially those with platinum-resistant or recurrent disease. Critical future priorities include developing standardised biomarkers for patient selection, establishing reliable methods to detect viral activity in tumour tissues, and understanding potential acquired resistance mechanisms. Given HCMV’s widespread prevalence and the urgent need for novel OC treatments, these targeted approaches offer significant potential for clinical translation.

### Limitations and Clinical Considerations

Discussion of the clinical feasibility and limitations of these emerging approaches is essential for evaluating their translational potential in OC treatment. TEDbodies and APECs were created to address key challenges associated with neoantigen-based therapies, which are highly personalised and face significant obstacles in antigen selection, manufacturing scalability, and cost-effectiveness. In contrast, HCMV-based approaches exploit the virus’ high seroprevalence in adults and characteristic memory inflation to target shared viral antigens across patients. This offers the potential for developing universal cancer vaccines rather than patient-specific neoantigen therapies, improving clinical accessibility and feasibility.

One limitation of TEDbodies is their requirement for tumour cells to express both the integrin ανβ5 and the very prevalent MHC-I variant, HLA-A*02:01 allele [151]. However, this could be addressed via the creation of other TEDbodies that are specific to other tumour markers for broader applicability. The role of the ανβ5 integrin remains contested in OC. Integrin ανβ5 has been implicated in multiple solid tumours [151,165,166], and Ruhi et al. [166] demonstrated its involvement in OC pathogenesis, showing that ανβ5 interacts with FAK and Akt pathways to assist in the protection against TRAIL-induced apoptosis by ovarian ascites. However, Nieberler et al. [165] suggest that while ανβ5 is expressed in other cancers, OC is primarily characterised with expression of other integrins such as ανβ6, ανβ3, and α5β1, which drive cell proliferation, tumour invasiveness and correlate with poor prognosis. Given these conflicting findings, comprehensive profiling of integrin expression patterns across OC subtypes is necessary to determine whether ανβ5-targeted TEDbodies are broadly applicable or whether alternative integrin-targeting strategies would be more effective.

Additionally, APECs address the HLA restriction limitation of TEDbodies. While TEDbodies require HLA-A*02:01 expression, combinatorial APECs (cAPECs) were engineered to target multiple HLA alleles, broadening patient eligibility [152]. However, critical limitations remain for both approaches. Current APEC and TEDbody strategies are restricted to patients with pre-existing anti-viral CD8+ T-cell responses. Expanding the repertoire of HCMV-derived epitopes that can bind to other HLA alleles is necessary to enable treatment of more diverse patient populations [151,152]. In addition, characterisation of integrin and MMP expression profiles across OC subtypes would be necessary to optimise APEC targeting and linker design. Critically, apart from Zhang et al.’s work with APECs [153], none of these HCMV-redirecting approaches have been comprehensively evaluated in OC preclinical models or clinical trials. The unique features of OC, such as ascites, platinum resistance mechanisms, and the distinct immunosuppressive TME, may significantly impact therapeutic efficacy. Disease-specific characterisation and optimisation are essential before clinical translation.

Usage of traditional anti-virals (such as gangiclovir and valganciclovir) for the treatment of HCMV is limited due to toxicity, resistance issues, and off-target effects [160]. There is also an important consideration to note, that to date there are no approved drugs for latent HCMV infection, only for lytic infection [160]. This could be particularly challenging if most HCMV-seropositive patients with OC are latently infected, and highlights the need to further uncover the viral profile of HCMV in OC. Letermovir and maribavir are novel anti-viral drugs which have been approved in recent years for treatment of lytic HCMV infection in organ transplant recipients and AIDS patients [160]. These drugs can be administered orally, which is more favourable to ensure patient compliance, and have been demonstrated to be less toxic than traditional anti-virals [160]. However, no comment can be made at present on whether it would be appropriate for the treatment of cancer as there is insufficient clinical evidence of its use in this context. Similarly, while HCMV vaccines are in development, significant barriers remain, including inadequate protection despite memory inflation, the virus’s immunoevasive capabilities, the unclear relationship between HCMV and immune responses, and the lack of suitable animal models [160]. Valganciclovir studies in GBM involved relatively small patient cohorts [163,164], and larger controlled trials are needed to definitively establish clinical benefit and determine optimal dosing, treatment duration, and patient selection criteria for cancer applications. Given that both GBM and OC are characterised by high recurrence rates and limited treatment options for relapsed disease, the promising safety profile of valganciclovir in GBM warrants investigation of similar strategies in OC, though OC-specific clinical validation is essential.

Antibody-based approaches (TEDbodies, APECs) require complex manufacturing processes and quality control systems. ACT/CAR-T therapies face scalability issues, high costs, and logistical challenges including patient leukapheresis, ex vivo manipulation, and cryopreservation [160,167,168]. For intra-tumoral injection approaches demonstrated in other tumours, accessing disseminated peritoneal disease or multiple metastatic sites in OC presents substantial practical barriers that would need to be addressed through alternative delivery methods or patient selection strategies [154].

Key questions remain unanswered for OC, specifically the following: (1) What proportion of OC patients have functional HCMV-specific memory T-cells capable of tumour recognition? (2) Will the immunosuppressive OC TME permit effective T-cell activation and tumour infiltration? (3) Can immune checkpoint molecules (PD-L1, HLA-E) that may be upregulated by HCMV counteract therapeutic T-cell responses? (4) What are the optimal combination strategies with existing OC therapies? (5) What are the risks of autoimmunity or off-target toxicity in the OC context?

Designing trials for HCMV-targeted OC therapy faces several challenges such as establishing appropriate endpoints (OS vs. PFS, etc.), developing methods to reliably stratify patients by HCMV status and viral load in tumour tissue, selecting optimal lines of therapy (frontline vs. recurrent/platinum-resistant disease), and defining biomarkers for patient selection and response monitoring. Overall, despite remaining in early-stage development with limited OC-specific data, these approaches demonstrate sufficient preclinical promise and translational feasibility to support their investigation as potential therapeutic strategies for OC. Priorities include comprehensive biomarker development and patient profiling, OC-specific preclinical validation in appropriate models, dose optimisation and safety studies, and carefully designed early-phase clinical trials in chosen populations.

## 7. Conclusions

The multifaceted role of HCMV in OC and other cancers is one of emerging research interest. As a result, many studies have suggested the possible classification of HCMV as an oncovirus. Primary HCMV infection may contribute to OC progression by various interconnected mechanisms such as immunomodulation of the TME, dysregulation of inflammatory signalling pathways, and strain-specific oncogenic effects. The identification of HCMV strain-specific effects on OC progression may give rise to potential therapeutic strategies such as the development of anti-virals against HCMV strains that could serve as a prognostic biomarker, helping to identify those at higher risk of aggressive disease progression. While anti-HCMV therapies are being explored for the treatment of GBM, to some clinical benefit, there is still a lack of focus in an OC context. A critical barrier to advancing HCMV research in OC is the current absence of established preclinical models for studying virus–tumour–immune interactions in this disease. To date, there are no studies investigating the productive infection of OC cells in vitro with HCMV. This presents a significant limitation as there is no standardised protocol that can be utilised to test different models of infection for OC, and to further explore the downstream effects of HCMV infection in the TME. To address this gap, a stepwise model approach is recommended. First, in vitro protocols should be established using HCMV-infected OC cell lines and patient-derived organoids to characterise viral tropism, replication kinetics, and direct tumour effects. Despite strain heterogeneity, a laboratory-adapted strain such as HCMV TB40/E should be used for consistency and potentially compared with a clinical isolate strain to assess biological relevance. Second, immune interactions should be studied via co-culture systems of infected cells with immune cells to validate the immunomodulatory mechanisms discussed in this review. Third, this could be advanced to in vivo models to establish physiological relevance, though this should be pursued after in vitro models have been optimised and validated. Developing these standardised, reproducible models will be critical for testing therapeutic strategies such as the synergy of combined anti-HCMV therapy with current gold-standard chemotherapies, and establishing HCMV’s role in OC pathogenesis. As HCMV’s role in OC continues to be elucidated through the implementation of appropriate model systems, potential novel markers may be identified and ultimately lead to improved diagnostic and therapeutic options for this fatal cancer.

## Figures and Tables

**Table 1:** Overview of the different classifications of HCMV strains (wild-type vs. clinical isolates vs. laboratory strains), and their known impacts in cancer.

	Prototype/Wild-Type	Clinical Isolate	Laboratory Strain
Common Strains	Merlin	HCMV-DB, HCMV-BL, VR1814, Toledo	AD169, Towne, TB40/E, and TB40/E-BAC4
Genomic Mutations	*RL13*, *UL128* [38,39]	*RL13*, *UL9*, *UL128*, *UL141* [38,39,40]	*RL13*, * ***UL128***, ***UL130***, ***UL131A***, *IRS1*, *US1*, *US2*, *UL40*, *UL1* [38,39]
Cellular Tropism and Replication	Closest match to clinical wild-type Broad cellular tropism [38]	Broad cellular tropism with macrophage preference Strong replication potential and transformative ability [16,39,41]	Growth restricted to fibroblasts with loss of broad tropism Widely used in research [38,39,42]
Oncogenic Potential	Limited data available, baseline for comparison with various strains	Activates oncogenic pathways (growth, survival, metastasis) Induces cellular transformation and EMT Causes epigenetic dysregulation Associated with tumour progression [13,16,21,32,39,40]	Induces genomic instability and DNA damage Promotes stem cell properties Associated with MET Mixed clinical outcomes (enhanced chemotherapy response vs. reduced GBM viability) [37,43,44,45]

* Genes in bold are important for viral entry into epithelial and endothelial cells [46]. EMT = epithelial-to-mesenchymal transition, MET = mesenchymal-to-epithelial transition, GBM = glioblastoma.

## Data Availability

Not applicable.

## Abbreviations

The following abbreviations are used in this manuscript:

5-LO	5-lipoxygenase
ACT	Adoptive cell therapy
Akt	Protein kinase B
APEC	Antibody-peptide epitope conjugates
BC	Breast cancer
BOT	Borderline ovarian tumour
CAR	Chimeric antigen receptor
CD90	Cluster of differentiation 90 (also known as Thy-1)
CmvIL-10	Cytomegalovirus-encoded human interleukin-10
COX-2	Cycloxygenase-2
CSC	Cancer stem cells
DC	Dendritic cell
DFS	Disease-free survival
EBV	Epstein–Barr virus
EMT	Epithelial-to-mesenchymal
EphA2	Ephrin receptor A2
ERK1/2	Extracellular signal-regulated kinases 1/2
EZH2	Enhancer of zeste homologue 2
gB	Envelope glycoprotein-B
GBM	Glioblastoma multiforme
gH	Envelope glycoprotein-H
GPCR	G protein-coupled receptor
HCMV	Human cytomegalovirus
HGSOC	High-grade serous ovarian cancer
HLA-E	Human leukocyte antigen-E
HPC	Hematopoietic stem and progenitor cells
HPV	Human papillomavirus
i.t	Intra-tumoural
ICB	Immune checkpoint blockade
IE1/2	Immediate early protein 1/2
IFN-γ	Interferon gamma
IL-10	Interleukin-10
IL-6	Interleukin-6
IRP	Immune risk profile
JAK	Janus-associated kinases
MDSC	Myeloid-derived suppressor cells
MET	Mesenchymal-to-epithelial
MHC-I	Major histocompatibility complex class I
MHC-II	Major histocompatibility complex class II
NF-κB	Nuclear factor-κB
NK	Natural killer cell
OC	Ovarian cancer
OCSC	Ovarian cancer stem cells
OS	Survival outcomes
PDGFRα	Platelet-derived growth factor receptor α
PD-1	Programmed cell death protein-1
PD-L1	Programmed death ligand-1
PFS	Progression-free survival
PGCC	Polyploid giant cancer cells
pp65	65 kDa tegument protein
pRB	Phosphorylated retinoblastoma protein
PRL	Prolactin
PRLR	Prolactin receptor
SC	Stem cell
SLC	Stem-like cells
STAT3	Signal transducer and activator of transcription 3
TAM	Tumour-associated macrophages
TAP	Peptide transporter associated with antigen processing
TCR	T-cell receptor
TEDbodies	T-cell epitope delivering antibodies
TEMRA	Terminally differentiated effector memory T-cells re-expressing CD45RA
TGF-β	Transforming growth factor-beta
Thy-1	Thy-1 cell surface antigen (also known as CD90)
TIL	Tumour-infiltrating lymphocytes
TLR2	Toll-like receptors 2
TME	Tumour microenvironment
TNF-α	Tumour necrosis factor-alpha
Treg	Regulatory T-cell
VEGF	Vascular endothelial growth factor
